# Plantar pressure in athletes with chronic ankle instability during single-leg landings at different heights

**DOI:** 10.3389/fbioe.2025.1702852

**Published:** 2025-12-16

**Authors:** Ansheng Wang, Qiang Zhang, Chengjun Li, Zhibo Tian, Xinxin Zhang, Yuliang Sun

**Affiliations:** 1 School of Physical Education, Shaanxi Normal University, Xi’an, China; 2 College of physical education and health, Guangxi Normal University, Guilin, China

**Keywords:** ankle injuries, landing, plantar pressure, athletes, injury risk

## Abstract

**Background:**

During landing, athletes with Chronic Ankle Instability (CAI) often display abnormal ankle joint movements, and changes occur as the height increases. There is a lack of sufficient research on assessing foot pressure distribution during landing at different heights for athletes with CAI, which would help determine their injury risk.

**Methods:**

Twenty male athletes with CAI and twenty healthy controls were recruited in a 2 (group: CAI vs. healthy) × 2 (height: 30 cm-vs. 40 cm) mixed experimental design. A 2 × 2 mixed-design ANOVA was used to evaluate the foot pressure distribution characteristics during landing, measured using a 400 × 400 mm FreeMed baropodometric platform.

**Results:**

Interaction effects were detected in peak force: metatarsal head 3 (MH3) (*p* = 0.047); load percentage: toes 2–5 (T2–5) (*p* = 0.050), MH3 (*p* = 0.038), rearfoot lateral (RF_L) (*p* = 0.045); peak pressure: MH3 (*p* = 0.013). Group effects were detected in peak force: T2–5 (*p* < 0.001), metatarsal head 4 (MH4) (*p* < 0.001), midfoot lateral (MF_L) (*p* < 0.001), and RF_L (*p* < 0.001); load percentage: MH4 (*p* < 0.001), MF_L (*p* < 0.001); peak pressure: T2–T5 (*p* = 0.001), MH4 (*p* < 0.001), MF_L (*p* < 0.001), and RF_L (*p* = 0.033); vCOP (*p* = 0.018). Pairwise comparisons showed that the peak force, pressure, and load distribution of athletes with CAI in T2–5, MH3, MH4, MF_L, and RF_L were significantly higher than those of the healthy group (*p* < 0.05). Additionally, the load percentage in RF_L and vCOP of athletes with CAI at a height of 40 cm was significantly greater than that of the healthy group (*p* < 0.05).

**Conclusion:**

Compared with healthy individuals, athletes with CAI have increased peak forces, pressures and load percentage at the T2–5, MH3, MH4, MF_L and RF_L during landing. The load percentage in RF_L and vCOP of athletes with CAI increases as the height increases, reflecting impaired postural control and a higher risk of re-injury. This highlights the need for trainers to design specific training programs based on the distribution characteristics of foot pressure during landing exercises.

## 1 Introduction

Ankle sprains are among the most common injuries in competitive sports (Newsham, 2019). It usually occurs during jumping and landing activities (Lytle et al., 2021). Over 40% of individuals with an ankle sprain are reported to develop chronic ankle instability (CAI) (Doherty et al., 2016). Athletes often face landing scenarios during training and competition, and those with CAI are more prone to control problems during landing, greatly raising their risk of re-injury. (Chan et al., 2022). Therefore, investigating the landing performance of athletes with CAI holds essential theoretical and practical significance.

The kinematics of CAI individual landing tasks are well understood (Chan et al., 2022). These studies show that CAI individuals typically display a greater eversion and plantar flexion angle during landing, raising their risk of injury (Lee et al., 2017; Simpson et al., 2019; Chan et al., 2022). However, few studies have examined the plantar pressure distribution of athletes with CAI during landing tasks. The survey of plantar pressure distribution explores the complex interactions between the plantar surface and the ground, and has become a vital focus in biomedical and health-related research (Abdul Razak et al., 2012). As an objective and precise analysis method, plantar pressure assessment has valuable applications in diagnosing and assessing ankle conditions, an important tool in clinical and research settings, which can help to improve the understanding of the motor function of CAI individuals (Arzehgar et al., 2025). Most current research on plantar pressure distribution in CAI cases is limited to flat gait activities like walking or running (Morrison et al., 2010; Koldenhoven et al., 2016; Wan et al., 2023). These studies indicate that athletes with CAI experience higher lateral foot pressure during walking and running, elevating the risk of re-sprain. Therefore, further research is needed to identify whether there are abnormal foot pressure patterns in athletes with CAI during landing.

Landing height plays an important role in an athlete’s landing performance; as the landing height varies, the biomechanical features of the lower limbs also change (Nordin and Dufek, 2017; Wang et al., 2021). Previous research has shown that as the landing height increases, the angular movements of the ankle, knee, and hip joints also increase, and the risk of lower limb injuries during landing rises (Nordin and Dufek, 2017). Additionally, a higher landing height may boost the speed of foot contact with the ground, which is more likely to cause injuries to the foot and ankle complex (Gribble et al., 2016). However, little is known about how foot pressure distribution varies in athletes with CAI under different landing heights.

This study used a 400 × 400 mm plantar pressure platform to analyze the characteristics of plantar pressure distribution in athletes with CAI when landing on one foot from different heights. We hypothesize that athletes with CAI experience greater lateral foot pressure and increased velocity of center of pressure (vCOP) during landing, which becomes more pronounced at greater heights.

## 2 Materials and methods

### 2.1 Participants

A group (CAI vs. Healthy) × height (30 cm vs. 40 cm) mixed-design ANOVA was used. Based on a previous study, using G*Power software (version 3.1.9.7, Heinrich Heine University Düsseldorf, Germany), with effect size (η_p_^2^) f = 0.25 (Lin et al., 2011), alpha = 0.05, power = 0.80, at least 34 participants were needed.

Participants’ characteristics are reported in Table 1. Forty male college athletes were recruited and evenly divided into CAI or Healthy control groups. The tested leg in healthy individuals (dominant or nondominant) corresponded to their matched CAI counterpart (Ross et al., 2009). Leg dominance was determined as the preferred leg for kicking a ball. Ethical approval was granted by the Specialized Committee on Scientific Ethics of the Academic Committee at Shaanxi Normal University (Approval No. 202516040), all procedures were conducted in accordance with the Declaration of Helsinki, and written informed consent was obtained from each participant before data collection. All participants voluntarily took part in the study and were students at the School of Physical Education, Shaanxi Normal University, each with at least 5 years of formal athletic training, including basketball, football, volleyball, long jump, high jump, sprinting (e.g., 100 m), and middle-distance running (e.g., 2000 m).

**TABLE 1:** Characteristics of participants CAI and Healthy.

Characteristics	CAI (n = 20)	Healthy (n = 20)
Age (yr)	21.90 ± 2.10	22.60 ± 1.70
Height (cm)	180.4 ± 8.2	178.4 ± 5.8
Weight (kg)	73.7 ± 7.4	71.4 ± 9.3
CAIT score	16.4 ± 3.7	27.2 ± 1.7
Dominant test limb, n	11	11
Nondominant test limb, n	9	9
Training duration (yr)	6.92 ± 1.44	7.36 ± 1.38

CAIT, Cumberland Ankle Instability Tool. Data are Mean ± SD.

Athletes with CAI were included based on the following criteria (Gribble et al., 2014): (1) The initial sprain to the ankle occurred at least 12 months before the experiment and was accompanied by inflammation symptoms (such as pain, swelling, etc.), resulting in at least 1 day of interrupted physical activity; (2) Recurrent ankle sprains on the same side occurring at least twice in the past 2 years; (3) Experiencing at least two episodes of ankle instability or “giving way” during daily physical activity within 6 months; (4) The Cumberland Ankle Instability Tool (CAIT) is a validated nine-item questionnaire used to assess the severity of functional ankle instability, with scores ranging from 0 to 30. Lower scores indicate greater instability. This study included individuals with a CAIT score of less than 24 as athletes with CAI (Hiller et al., 2006).

Healthy athletes were included based on the following criteria: (1) no history of ankle sprain causing at least 1 day off from physical activity; (2) CAIT score above 24.

Participants were excluded based on the following criteria: (1) having flat feet or high arches; (2) experiencing acute sports-related injuries or chronic conditions such as limb osteoarthritis or neuromuscular disorders; and (3) having a history of lower extremity injuries other than unilateral CAI.

### 2.2 Apparatus and measurement

The equipment used in this study included a 400 × 400 mm FreeMed baropodometric platform (Sensor Medica, Inc., Via Umberto Agnelli 11, Guidonia Montecelio, Rome, Italy). The FREEMED model is a lightweight, portable platform. This study uses a real-time sampling frequency of 400 Hz and automatically sets up and calibrates with 10-bit resolution. All sensors are resistive, coated with 24K gold, and made of conductive rubber. This device can accurately identify the plantar pressure in each sole area and has been fully verified (Długosz-Boś et al., 2021; Qian et al., 2021; Rdzanek et al., 2022; Elabd et al., 2023; Feng et al., 2023). A wooden jump box (30 cm × 35 cm × 40 cm) was used in the experiment. The 30 cm height was chosen based on a prior study indicating it offers optimal biomechanical benefits during single-legged landing (Wang and Peng, 2013). The 40 cm height was selected to increase task intensity while moderately reducing injury risks, as some participants reported discomfort with higher drop heights during pretesting.

**FIGURE 1:**
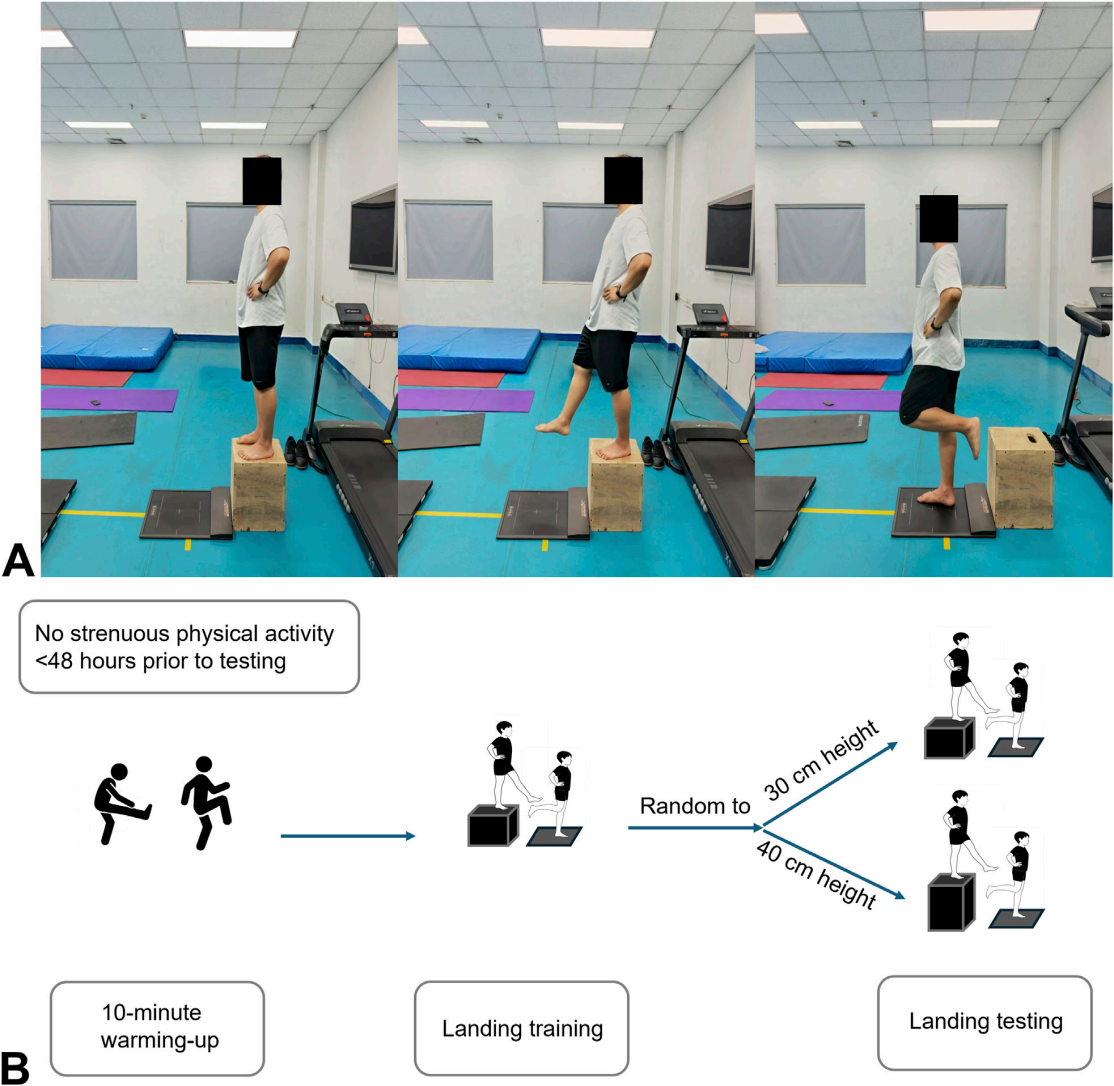
**(A)** Experiment environment and landing task **(B)** experimental procedure.

Before data collection, participants completed a guided full-body warm-up lasting about 10 min. They were then asked to remove their shoes and perform barefoot single-leg drop landings to familiarize themselves with the procedure, as barefoot conditions were used to avoid the potential influence of footwear on the accurate plantar pressure distribution in athletes with CAI. The platform was positioned on level ground. During data collection, participants stood still on the jump box (30 cm or 40 cm) with hands on their hips, extended one foot forward, and executed a single-leg landing onto the platform (Figure 1A). A 60-s rest separated each jump. Each participant was randomly tested at 30 cm or 40 cm height to avoid the order effect. The experimental procedure is shown in Figure 1B.

### 2.3 Data analysis

**FIGURE 2:**
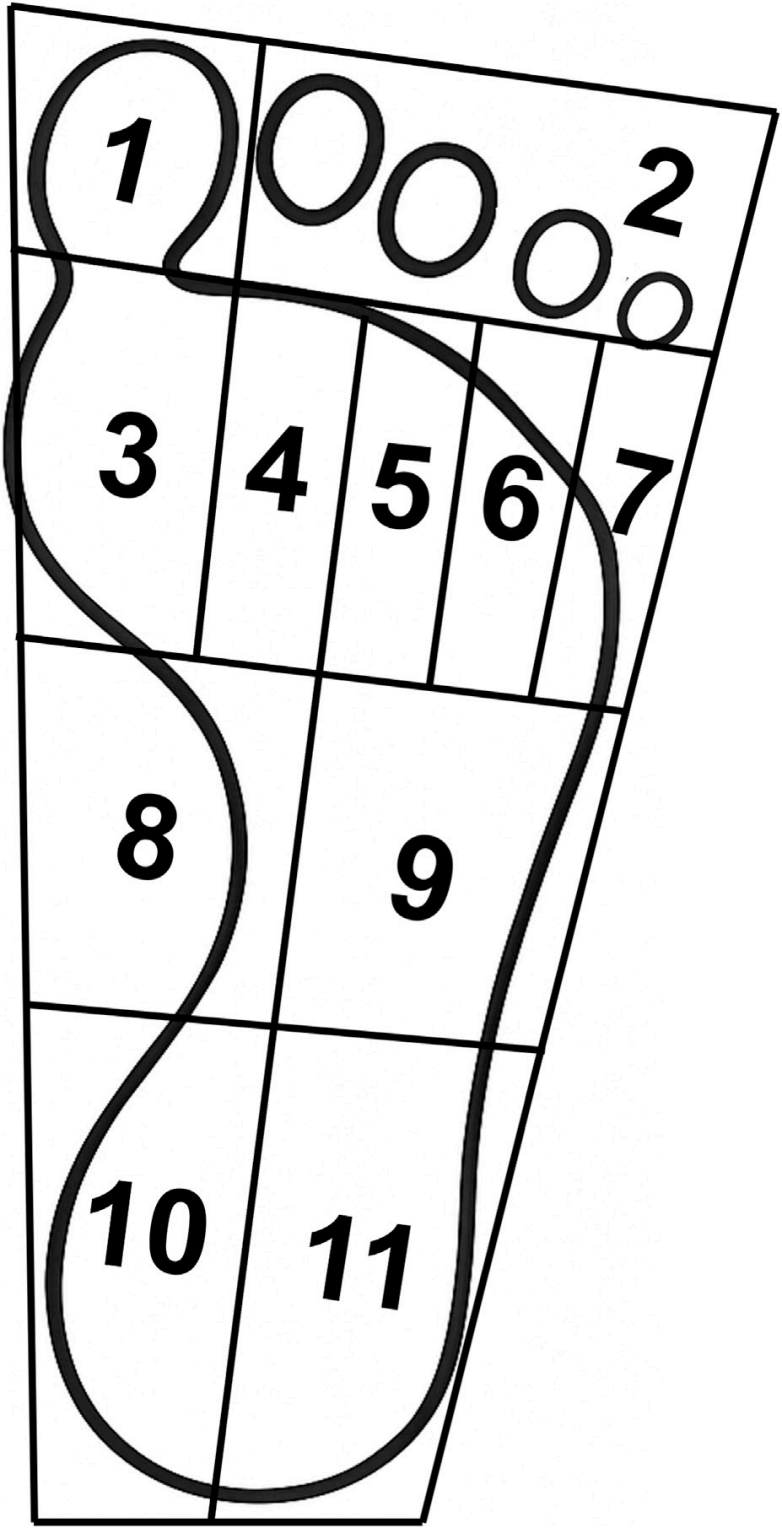
Definition of Foot Pressure Distribution. Division of plantar regions: (1) toe 1, (2) toes 2–5, (3) metatarsal head 1, (4) metatarsal head 2, (5) metatarsal head 3, (6) metatarsal head 4, (7) metatarsal head 5, (8) midfoot medial, (9) midfoot lateral, (10) rearfoot medial, and (11) rearfoot lateral.

FreeStep (v1.6.005, Sensor Medica, Roma, Italy) automatically evaluates plantar pressure without shoes. The FREESTEP software divides the footprint into 11 zones: the (1) toe 1, (2) toes 2–5, (3) metatarsal head 1, (4) metatarsal head 2, (5) metatarsal head 3, (6) metatarsal head 4, (7) metatarsal head 5, (8) midfoot medial, (9) midfoot lateral, (10) rearfoot medial, and (11) rearfoot lateral (Figure 2). This standardized regional division has been widely adopted in previous studies and has been proven to be reliable and effective for the analysis of foot pressure (Qian et al., 2021; Elabd et al., 2023). To comprehensively capture the biomechanical differences in different areas of the CAI individual’s foot sole, and to conduct a detailed analysis of the specific change characteristics in each area, this study selected all 11 preset regions of the system and conducted analyses on each region. Since the critical risk period for ankle sprains usually occurs within the first 200 ms after landing—when peak ankle inversion typically occurs (Terada and Gribble, 2015)—the landing event was defined as the time when the baropodometric platform first detected foot–ground contact, and the subsequent 200 ms were used for analysis. Each participant completed five valid trials at each height. FREESTEP software exports the data for each participant into a CSV file. The exported data were then used to identify the Peak Force, Peak Pressure in the 11 regions (normalized by body weight), Load Percentage, and the vCOP. The result was calculated based on the average of the five data trials.

### 2.4 Statistical analysis

All analyses were done with IBM SPSS Statistics software (SPSS Statistics v27.0, IBM Corp., US). Data for continuous variables are presented as mean ± SD (Mean ± SD). The Shapiro-Wilk tests were used to determine the normality of all outcome variables, and Levene’s test supported the assumption of variance homogeneity. To evaluate the main and interaction effects of group (CAI vs. Healthy) and height (30 cm vs. 40 cm), a 2 × 2 mixed-design ANOVA was used. When significant main effects were present, pairwise comparisons were conducted with Bonferroni adjustment. Simple effect analyses were carried out when interaction effects were significant, and subsequent comparisons were corrected using the Bonferroni method. All statistical tests were two-sided, with a significance level set at α = 0.05. Statistically significant p-values were reported accordingly. According to Cohen’s guidelines, η_p_^2^ values of 0.01, 0.06, and 0.14 were interpreted as small, medium, and large effects, respectively.

## 3 Results

### 3.1 Peak force

Significant interaction effects were observed in MH3 (*p* = 0.047) and MH5 (*p* = 0.049). Specifically, the CAI group demonstrated greater value than the healthy group at 30 cm in MH3 (*p* < 0.001). In MH5, the CAI group had a significantly lower Peak Force than the healthy group at 30 cm (*p* = 0.005). Significant group main effects were observed in the CAI group across the following regions: T2–T5 (*p* < 0.001), MH4 (*p* < 0.001), MF_M (*p* = 0.010), MF_L (*p* < 0.001), RF_M (*p* < 0.001), and RF_L (*p* < 0.001). Additionally, a main effect of height was observed in RF_M (*p* = 0.002). At both heights, the CAI group showed greater value than the healthy group in T2–T5, MH4, and RF_L. In contrast, the CAI group showed lower values at MF_M and RF_M. Notably, the two groups’ value in RF_M decreased with increasing height. Full statistical outcomes are presented in Table 2 and Supplementary Material (Supplementary Table S1).

**TABLE 2:** Statistical results from the ANOVAs comparing the Peak Force (normalized by body weight).

PF/BW (N/kg)	CAI (n = 20)		Healthy (n = 20)		Group	Height	Group*Height
	30 cm	40 cm	30 cm	40 cm	*p* value		
T1	0.042 ± 0.031	0.040 ± 0.037	0.052 ± 0.031	0.052 ± 0.034	0.303	0.619	0.600
T2–T5	0.042 ± 0.033^a^ ^b^	0.042 ± 0.033	0.023 ± 0.018	0.021 ± 0.015	<0.001	0.777	0.824
MH1	0.127 ± 0.044	0.128 ± 0.036	0.143 ± 0.044	0.139 ± 0.034	0.057	0.841	0.702
MH2	0.076 ± 0.037	0.072 ± 0.041	0.081 ± 0.031	0.084 ± 0.036	0.194	0.920	0.481
MH3	0.080 ± 0.054^a^	0.068 ± 0.050	0.044 ± 0.032	0.060 ± 0.039	0.004	0.796	0.047
MH4	0.098 ± 0.026^a^ ^b^	0.095 ± 0.036	0.057 ± 0.030	0.060 ± 0.029	<0.001	0.958	0.560
MH5	0.085 ± 0.046^a^	0.105 ± 0.051	0.114 ± 0.040	0.102 ± 0.043	0.053	0.615	0.049
MF_M	0.211 ± 0.043^a^	0.220 ± 0.059	0.240 ± 0.047	0.236 ± 0.045	0.010	0.375	0.708
MF_L	0.209 ± 0.054^b^	0.212 ± 0.033	0.186 ± 0.046	0.178 ± 0.046	<0.001	0.783	0.463
RF_M	0.089 ± 0.051^a^ ^b^ ^c^ ^d^	0.057 ± 0.024	0.134 ± 0.058	0.110 ± 0.051	<0.001	0.002	0.676
RF_L	0.089 ± 0.057^a^ ^b^	0.111 ± 0.068	0.053 ± 0.028	0.055 ± 0.034	<0.001	0.149	0.253

T1, toe 1; T2–T5, toes 2–5; MH1, metatarsal head 1; MH2, metatarsal head 2; MH3, metatarsal head 3; MH4 metatarsal head 4; MH5, metatarsal head 5; MF_M, midfoot medial; MF_L, midfoot lateral; RF_M, rearfoot medial; RF_L, rearfoot lateral.

^a^ significant difference between groups (30 cm).

^b^ significant difference between groups (40 cm).

^c^ significant difference between 30 cm and 40 cm within the Healthy group.

^d^ significant difference between 30 cm and 40 cm within the CAI group.

### 3.2 Load percentage

Significant interaction effects were observed in the T2–T5 (*p* = 0.050), MH3 (*p* = 0.038), and RF_L (*p* = 0.045) regions: In the T2–T5, the CAI group was higher than the healthy group at 40 cm (*p* < 0.001); in the MH3, the CAI group was higher than the healthy group at 30 cm (*p* < 0.001); in the RF_L, the CAI group’s value significantly increased at 40 cm (*p* = 0.001), and it was higher than the healthy group at both heights (30 cm: *p* < 0.001, 40 cm: *p* < 0.001). Significant group main effects were observed in the CAI group across the following regions: MH4 (*p* < 0.001), MH5 (*p* = 0.005), MF_M (*p* < 0.001), MF_L (*p* < 0.001), and RF_M (*p* < 0.001). The CAI group had significantly higher values at both heights compared to the healthy group in MH4 and MF_L; the CAI group had significantly lower values than the healthy group at both heights in MF_M and RF_M; the CAI group’s value at 30 cm was lower than that of the healthy group in MH5. Full statistical outcomes are presented in Table 3 and Supplementary Material (Supplementary Table S2).

**TABLE 3:** Statistical results from the ANOVAs comparing the Load percentage.

Load percentage %	CAI (n = 20)		Healthy (n = 20)		Group	Height	Group*Height
	30 cm	40 cm	30 cm	40 cm	p value		
T1	3.77 ± 2.47	3.25 ± 2.81	3.69 ± 2.59	3.27 ± 2.35	0.953	0.206	0.890
T2–T5	2.29 ± 1.75^b^	2.90 ± 2.39	1.78 ± 1.35	1.39 ± 1.07	<0.001	0.665	0.050
MH1	10.62 ± 3.89	11.32 ± 4.60	12.24 ± 3.74	11.93 ± 3.97	0.092	0.783	0.477
MH2	7.34 ± 4.25	6.77 ± 4.53	7.07 ± 3.36	7.5 ± 3.76	0.746	0.911	0.418
MH3	7.07 ± 4.85^a^	5.72 ± 4.95	3.29 ± 3.00	4.59 ± 3.30	<0.001	0.968	0.038
MH4	7.84 ± 3.17^a^ ^b^	7.37 ± 3.40	4.81 ± 3.10	4.58 ± 2.36	<0.001	0.457	0.803
MH5	7.41 ± 4.19^a^ ^c^	7.23 ± 3.49	10.42 ± 4.48	8.62 ± 4.72	0.005	0.175	0.262
MF_M	18.38 ± 5.05^a^ ^b^	18.47 ± 6.64	23.34 ± 5.71	22.77 ± 6.22	<0.001	0.805	0.733
MF_L	19.30 ± 4.82^a^ ^b^	20.68 ± 4.58	15.96 ± 4.83	15.52 ± 4.43	<0.001	0.553	0.258
RF_M	5.62 ± 3.63^a^ ^b^	4.16 ± 2.77	9.50 ± 5.37	8.39 ± 4.78	<0.001	0.072	0.802
RF_L	5.61 ± 2.98^a^ ^b^ ^d^	8.83 ± 6.08	3.44 ± 2.21	4.25 ± 3.07	<0.001	<0.001	0.045

T1, toe 1; T2–T5, toes 2–5; MH1, metatarsal head 1; MH2, metatarsal head 2; MH3, metatarsal head 3; MH4 metatarsal head 4; MH5, metatarsal head 5; MF_M, midfoot medial; MF_L, midfoot lateral; RF_M, rearfoot medial; RF_L, rearfoot lateral.

^a^ significant difference between groups (30 cm).

^b^ significant difference between groups (40 cm).

^c^ significant difference between 30 cm and 40 cm within the Healthy group.

^d^ significant difference between 30 cm and 40 cm within the CAI, group.

### 3.3 Peak pressure

Significant interaction was observed in the MH3 (*p* = 0.013): The CAI group was significantly higher than the healthy group at 30 cm (*p* < 0.001). Significant group main effects were observed in the CAI group across the following regions: T2–T5 (*p* = 0.001), MH1 (*p* = 0.001), MH4 (*p* < 0.001), MF_M (*p* < 0.001), MF_L (*p* < 0.001), RF_M (*p* = 0.007), and RF_L (*p* = 0.033). In the MH4 and MF_L, CAI group’s value was higher than that of the healthy group at both heights; in the T2–T5 at 30 cm and in the RF_L at 40 cm, CAI group’s value was higher than that of the healthy group; while in the MH1, MF_M and RF_M at 30 cm, CAI group’s value was lower than that of the healthy group. Full statistical outcomes are presented in Table 4 and Supplementary Material (Supplementary Table S3).

**TABLE 4:** Statistical results from the ANOVAs comparing the Peak pressure (normalized by body weight).

PP/BW (kPa/kg)	CAI (n = 20)		Healthy (n = 20)		Group	Height	Group*Height
	30 cm	40 cm	30 cm	40 cm	p value		
T1	0.586 ± 0.294	0.631 ± 0.192	0.620 ± 0.190	0.636 ± 0.222	0.591	0.434	0.706
T2–T5	0.699 ± 0.257^a^	0.561 ± 0.305	0.473 ± 0.228	0.487 ± 0.262	0.001	0.155	0.083
MH1	0.701 ± 0.143^a^	0.768 ± 0.190	0.837 ± 0.124	0.822 ± 0.170	0.001	0.293	0.098
MH2	0.806 ± 0.183	0.778 ± 0.245	0.802 ± 0.202	0.810 ± 0.230	0.715	0.769	0.595
MH3	0.827 ± 0.293^a^ ^c^	0.739 ± 0.335	0.495 ± 0.239	0.636 ± 0.289	<0.001	0.557	0.013
MH4	0.938 ± 0.227^a^ ^b^	0.633 ± 0.192	0.899 ± 0.232	0.670 ± 0.209	<0.001	0.981	0.273
MH5	0.822 ± 0.190	0.846 ± 0.124	0.809 ± 0.148	0.834 ± 0.136	0.614	0.335	0.970
MF_M	0.749 ± 0.127^a^	0.774 ± 0.151	0.873 ± 0.160	0.842 ± 0.152	<0.001	0.904	0.279
MF_L	0.881 ± 0.154^a^ ^b^	0.915 ± 0.160	0.785 ± 0.121	0.795 ± 0.134	<0.001	0.296	0.577
RF_M	0.576 ± 0.167^a^	0.640 ± 0.192	0.684 ± 0.166	0.686 ± 0.167	0.007	0.285	0.309
RF_L	0.660 ± 0.151^b^	0.742 ± 0.233	0.624 ± 0.161	0.634 ± 0.174	0.033	0.096	0.189

T1, toe 1; T2–T5, toes 2–5; MH1, metatarsal head 1; MH2, metatarsal head 2; MH3, metatarsal head 3; MH4 metatarsal head 4; MH5, metatarsal head 5; MF_M, midfoot medial; MF_L, midfoot lateral; RF_M, rearfoot medial; RF_L, rearfoot lateral.

^a^ significant difference between groups (30 cm).

^b^ significant difference between groups (40 cm).

^c^ significant difference between 30 cm and 40 cm within the Healthy group.

^d^ significant difference between 30 cm and 40 cm within the CAI, group.

### 3.4 Velocity of COP

**FIGURE 3:**
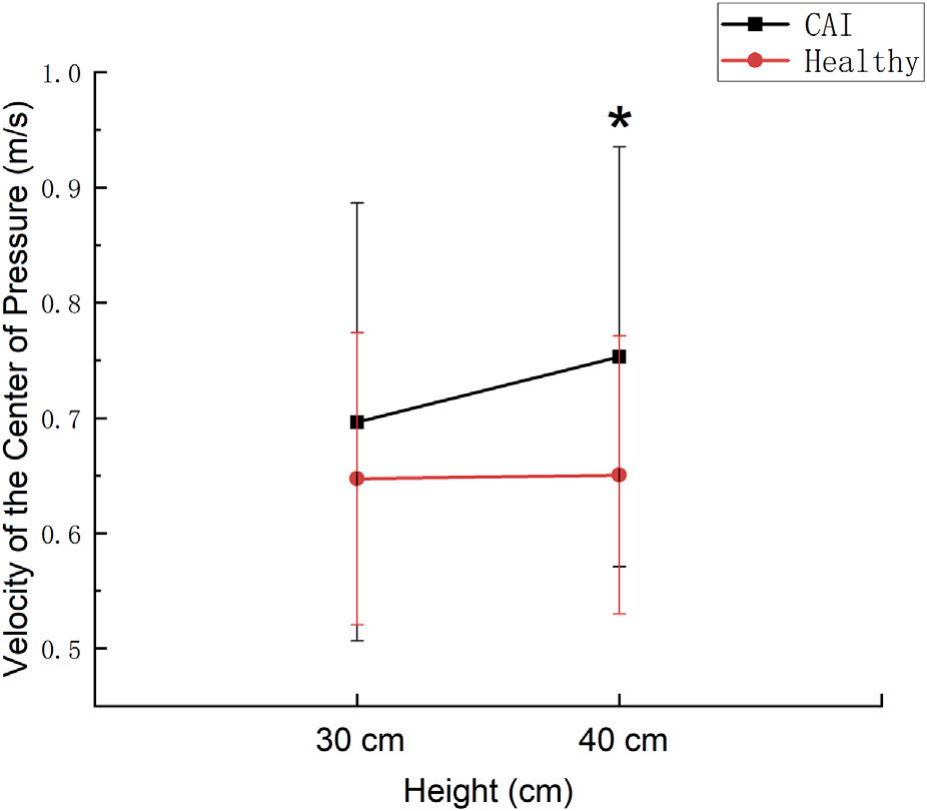
The velocity of the Center of Pressure at 30 cm and 40 cm in the CAI and Healthy groups. * Indicates a significant difference between the two groups.

The vCOP exhibited a significant group main effect (*p* = 0.018), with the CAI group showing a significantly higher velocity at 40 cm than the healthy group (Figure 3).

## 4 Discussion

This study employed a 400 × 400 mm foot pressure measurement platform to analyze the characteristics of the foot pressure distribution of athletes with CAI when they landed on one foot at a distance of 30 or 40 cm. athletes with CAI showed higher Peak Force, Load Percentage, and Peak Pressure in specific regions (T 2-3-4-5, MH3, MH4, MF_L, RF_L) when landing from 30 cm to 40 cm. At 40 cm, athletes with CAI had higher vCOP. Athletes with CAI Peak Force in RF_M decreased as height increased, while Load Percentage in RF_L increased. The findings essentially confirmed the hypothesis. Almost all outcomes’ η_p_^2^ were medium or large, further supporting these findings’ significance.

Our results showed that athletes with CAI exhibited an abnormal plantar pressure distribution pattern during landing manoeuvres, characterized by a significant increase in lateral foot pressure, force, and load percentage. This further supports previous observations of abnormal plantar pressure distribution in CAI individuals (Morrison et al., 2010; Koldenhoven et al., 2016; Wan et al., 2023). The greater load and pressure on the side of the foot may cause frequent ankle sprains (Hawrylak et al., 2021). During competitive sports, Unilateral landing tasks are a common and important movement (Dufek and Bates, 1991). It is a high-impact and injury-prone dynamic action that imposes large and rapid impulse loads on the ankle, which can initiate the mechanism of a lateral ankle sprain (Hertel, 2002). This study chose a dynamic unilateral landing task of variable height to demonstrate that abnormal plantar pressure distribution in CAI is not only present in static or low-load tasks, but also persists and may be exacerbated during high-demand, movement-specific actions.

Evidence indicates that most ankle sprains occur when the joint is positioned in inversion (Xu et al., 2022). In this posture, the ankle ligaments become highly susceptible to injury (Fong et al., 2007; Hubbard, 2008). These findings align with those of Woods et al., who observed high rates of ankle and lower leg lateral ligament injuries, potentially caused by habitual loading on the foot’s lateral side (Woods et al., 2002). Recurrent sprains may lead to ankle ligament laxity (Kobayashi et al., 2024). The increased lateral pressure during landing may exert excessive tensile stress on the ligament, especially when the ankle is varus. Given the possible ligament relaxation of CAI individuals themselves (Kobayashi et al., 2024), this pressure distribution change suggests that landing conditions at different heights may exacerbate the vulnerability of the lateral ligament complex.

At the same time, landing requires high demands on muscle control (Beutler, 2009). The peroneal muscle group is indispensable in controlling foot eversion (Hopkins et al., 2009); the peroneal muscle regulates rapid and excessive supination of the ankle complex, critical in stabilizing the dynamic joint and protecting against ankle varus injury (Santilli et al., 2005; Ziai et al., 2013). However, previous evidence has confirmed that CAI individuals exhibit delayed PL activation during dynamic activities and reduced muscle activity compared to healthy individuals (Flevas et al., 2017). This may lead to reduced inversion resistance (Kobayashi et al., 2024). Previous research on three-dimensional motion analysis shows that CAI patients have a greater inversion angle during landing than healthy individuals (Herb et al., 2018; Kim et al., 2019). Meanwhile, a recent study suggests that individuals with CAI adopt a strategy to increase ankle dorsiflexion angle when landing from medium and high heights (Zhang et al., 2024), which may cause a further shift in plantar pressure to the hindfoot. It can be inferred that athletes with CAI direct more pressure and contact to the lateral foot simultaneously with the increased ankle varus tendency when landing, resulting in higher lateral foot pressure. At the same time, with the increase in height, more pressure shifted to the hindfoot. This increases the risk of the CAI athlete suffering re-injury upon landing.

As external pressure on the foot increases, we observe that as the height increases, the vCOP of athletes with CAI when landing from the ground significantly accelerates and is much greater than that of healthy individuals. Previous studies have confirmed that athletes with CAI have impaired proprioception of the ankle joint, which affects the movement of the ankle joint during landing and increases the risk of ankle inversion injury (Wang et al., 2025). Moreover, as the landing height increases, the proprioception of athletes with CAI decreases (Kang et al., 2022). This may result in the inability of CAI individuals to maintain controlled weight transfer when landing from a higher height and further decrease postural stability, resulting in increased vCOP.

Some studies have indicated that accelerated COP movement suggests a decrease in the posture stability of athletes with CAI, possibly due to differences in muscle strength (Wan et al., 2023). Moreover, the external rotation strength of the ankle joint of athletes with CAI may decline, causing them to be unable to control the eversion of the ankle joint better, thereby preventing internal sprains. Additionally, through the measurement indicators of COP movement speed, namely, posture control, potentially due to differences in muscle strength (Wan et al., 2023). Additionally, aspects of neuromuscular control can be quantified through measures of postural control (Gribble et al., 2004). Therefore, it can be inferred that when athletes with CAI land from a height, their neuromuscular control ability significantly decreases, further increasing the risk of re-injury. This deterioration in postural control likely reflects a progressive decline in neuromuscular coordination as landing height increases in athletes with CAI. Accordingly, it can be inferred that athletes with CAI experience significantly impaired neuromuscular control during high-impact landings, which further elevates their risk of re-injury.

The study reveals that athletes with CAI exhibit altered plantar pressure patterns, heightening the risk of lateral ankle sprains. This abnormal pressure, combined with higher vCOP, is likely due to delayed peroneal muscle activation and weaker neuromuscular control (Santilli et al., 2005; Ziai et al., 2013). Based on these findings, future training programs should enhance muscle strength, particularly in the peroneal group, to improve ankle stability and postural control. Previous studies have demonstrated that training the foot’s intrinsic muscles can significantly promote functional recovery and enhance neuromuscular control in individuals with CAI (Hoch et al., 2023). On this basis, if proprioceptive training and foot muscle function training can be combined with the abnormal characteristics of plantar pressure distribution (Tang et al., 2020), to provide a more comprehensive intervention programme. CAI athletes should choose appropriate external assistance according to different sports and training situations to better cope with the dynamic demands of high-impact landing tasks and reduce the risk of re-injury.

Limitations of this study include the following: (1) This study included only male athlete participants and a relatively small sample size, which may limit the generalizability of the findings across genders, to non-athletic populations, and to broader cohorts. (2) The three-dimensional motion capture system was not used due to equipment limitations. It is suggested that the two systems be combined in future experiments to better reflect the individual’s overall performance. (3) Due to the inherent fragility of the ankle joints in athletes with CAI, an excessively high landing height was not chosen. This might not meet the requirements of certain specific sports scenarios. (4) The absence of footwear during testing may impact ankle stability and affect the generalizability of the findings to real-world conditions. (5) Landing on flat ground is not a typical scenario in which sprains occur and may limit the generalizability of this study; landing on a floatable plate could be attempted in future studies (Zhu et al., 2025).

## 5 Conclusion

Results show that compared with healthy individuals, athletes with CAI have increased peak forces, pressures and load percentage at the T2–5, MH3, MH4, MF_L and RF_L during landing. Reveal the plantar load pattern of athletes with CAI shifted from the medial to the lateral area. The load percentage in RF_L and vCOP of athletes with CAI increases as the height increases, reflecting impaired postural control and a higher risk of re-injury. This highlights the need for trainers to design specific training programs based on the distribution characteristics of foot pressure during landing exercises.

## Data Availability

The raw data supporting the conclusions of this article will be made available by the authors, without undue reservation.
