# Correction: Plantar pressure in athletes with chronic ankle instability during single-leg landings at different heights

**DOI:** 10.3389/fbioe.2025.1773746

**Published:** 2026-01-09

**Authors:** Ansheng Wang, Qiang Zhang, Chengjun Li, Zhibo Tian, Xinxin Zhang, Yuliang Sun

**Affiliations:** 1 School of Physical Education, Shaanxi Normal University, Xi’an, China; 2 College of Physical Education and Health, Guangxi Normal University, Guilin, China

**Keywords:** ankle injuries, landing, plantar pressure, athletes, injury risk

There was a mistake in Table 2 as published. The standard deviation for one of the values in the Table (T1, in the CAI group at 30 cm) was inadvertently omitted. The corrected Table 2 appears below.

**TABLE 2:** Statistical results from the ANOVAs comparing the Peak Force (normalized by body weight).

PF/BW (N/kg)	CAI (n = 20)		Healthy (n = 20)		Group	Height	Group*Height
	30 cm	40 cm	30 cm	40 cm	*p* value		
T1	0.042 ± 0.031	0.040 ± 0.037	0.052 ± 0.031	0.052 ± 0.034	0.303	0.619	0.600
T2–T5	0.042 ± 0.033^a^ ^b^	0.042 ± 0.033	0.023 ± 0.018	0.021 ± 0.015	<0.001	0.777	0.824
MH1	0.127 ± 0.044	0.128 ± 0.036	0.143 ± 0.044	0.139 ± 0.034	0.057	0.841	0.702
MH2	0.076 ± 0.037	0.072 ± 0.041	0.081 ± 0.031	0.084 ± 0.036	0.194	0.920	0.481
MH3	0.080 ± 0.054^a^	0.068 ± 0.050	0.044 ± 0.032	0.060 ± 0.039	0.004	0.796	0.047
MH4	0.098 ± 0.026^a^ ^b^	0.095 ± 0.036	0.057 ± 0.030	0.060 ± 0.029	<0.001	0.958	0.560
MH5	0.085 ± 0.046^a^	0.105 ± 0.051	0.114 ± 0.040	0.102 ± 0.043	0.053	0.615	0.049
MF_M	0.211 ± 0.043^a^	0.220 ± 0.059	0.240 ± 0.047	0.236 ± 0.045	0.010	0.375	0.708
MF_L	0.209 ± 0.054^b^	0.212 ± 0.033	0.186 ± 0.046	0.178 ± 0.046	<0.001	0.783	0.463
RF_M	0.089 ± 0.051^a^ ^b^ ^c^ ^d^	0.057 ± 0.024	0.134 ± 0.058	0.110 ± 0.051	<0.001	0.002	0.676
RF_L	0.089 ± 0.057^a^ ^b^	0.111 ± 0.068	0.053 ± 0.028	0.055 ± 0.034	<0.001	0.149	0.253

T1, toe 1; T2–T5, toes 2–5; MH1, metatarsal head 1; MH2, metatarsal head 2; MH3, metatarsal head 3; MH4 metatarsal head 4; MH5, metatarsal head 5; MF_M, midfootmedial; MF_L, midfoot lateral; RF_M, rearfoot medial; RF_L, rearfoot lateral.

^a^ significant difference between groups (30 cm).

^b^ significant difference between groups (40 cm).

^c^ significant difference between 30 cm and 40 cm within the Healthy group.

^d^ significant difference between 30 cm and 40 cm within the CAI, group.

The original article has been updated.

