# Supplementary material for: Plantar pressure in athletes with chronic ankle instability during single-leg landings at different heights
Source: Front Bioeng Biotechnol. 2025 Dec 16;13:1702852. doi: 10.3389/fbioe.2025.1702852 (PMC12748167; doi:10.3389/fbioe.2025.1702852)
Supplement: Supplementary file 1 [file Table1.docx]

Supplementary Material

| **Region** | **Group***  **Height** | | | **Height** | | | **Group** | | |
| --- | --- | --- | --- | --- | --- | --- | --- | --- | --- |
|  | **F (*1, 38*)** | ***p* value** | ***η_p_²*** | **F (*1, 38*)** | ***p* value** | ***η_p_²*** | **F (*1, 38*)** | ***p* value** | ***η_p_²*** |
| T1 | 0.278 | 0.600 | 0.004 | 0.250 | 0.619 | 0.003 | 1.074 | 0.303 | 0.014 |
| T 2-3-4-5 | 0.050 | 0.824 | 0.001 | 0.081 | 0.777 | 0.001 | 26.173 | **<0.001** | 0.261 |
| MH1 | 0.147 | 0.702 | 0.002 | 0.040 | 0.841 | 0.001 | 3.736 | 0.057 | 0.048 |
| MH2 | 0.501 | 0.481 | 0.007 | 0.010 | 0.920 | 0.000 | 1.720 | 0.194 | 0.023 |
| MH3 | 4.071 | **0.047** | 0.052 | 0.067 | 0.796 | 0.001 | 9.032 | **0.004** | 0.109 |
| MH4 | 0.342 | 0.560 | 0.005 | 0.003 | 0.958 | 0.000 | 50.248 | **<0.001** | 0.404 |
| MH5 | 3.999 | **0.049** | 0.051 | 0.255 | 0.615 | 0.003 | 3.874 | 0.053 | 0.050 |
| MF M | 0.141 | 0.708 | 0.002 | 0.797 | 0.375 | 0.011 | 6.774 | **0.010** | 0.086 |
| MF L | 0.544 | 0.463 | 0.007 | 0.077 | 0.783 | 0.001 | 14.264 | **<0.001** | 0.162 |
| RF M | 0.176 | 0.676 | 0.002 | 10.685 | **0.002** | 0.126 | 35.189 | **<0.001** | 0.322 |
| RF L | 1.325 | 0.253 | 0.018 | 2.130 | 0.149 | 0.028 | 42.265 | **<0.001** | 0.364 |

Table S1. Results of mixed-design ANOVA of Peak Force

T 1, toe 1; T 2-3-4-5, toes 2-3-4-5; MH1, metatarsal head 1; MH2, metatarsal head 2; MH3, metatarsal head 3; MH4 metatarsal head 4; MH5, metatarsal head 5; MF M, midfoot medial; MF L, midfoot lateral; RF M, rearfoot medial; RF L, rearfoot lateral.

Table S2. Results of mixed-design ANOVA of Load Percentage

| **Region** | **Group***  **Height** | | | **Height** | | | **Group** | | |
| --- | --- | --- | --- | --- | --- | --- | --- | --- | --- |
|  | **F (*1, 38*)** | ***p* value** | ***η_p_²*** | **F (*1, 38*)** | ***p* value** | ***η_p_²*** | **F (*1, 38*)** | ***p* value** | ***η_p_²*** |
| T1 | 0.019 | 0.890 | 0.000 | 1.626 | 0.206 | 0.021 | 0.004 | 0.953 | 0.000 |
| T 2-3-4-5 | 3.968 | **0.050** | 0.051 | 0.189 | 0.665 | 0.003 | 12.808 | **<0.001** | 0.148 |
| MH1 | 0.510 | 0.477 | 0.007 | 0.077 | 0.783 | 0.001 | 2.915 | 0.092 | 0.038 |
| MH2 | 0.664 | 0.418 | 0.009 | 0.013 | 0.911 | 0.000 | 0.105 | 0.746 | 0.001 |
| MH3 | 4.464 | **0.038** | 0.057 | 0.002 | 0.968 | 0.000 | 13.052 | **<0.001** | 0.150 |
| MH4 | 0.063 | 0.803 | 0.001 | 0.559 | 0.457 | 0.007 | 30.604 | **<0.001** | 0.293 |
| MH5 | 1.277 | 0.262 | 0.017 | 1.872 | 0.175 | 0.025 | 8.482 | **0.005** | 0.103 |
| MF M | 0.117 | 0.733 | 0.002 | 0.061 | 0.805 | 0.001 | 19.709 | **<0.001** | 0.210 |
| MF L | 1.298 | 0.258 | 0.017 | 0.355 | 0.553 | 0.005 | 29.398 | **<0.001** | 0.284 |
| RF M | 0.063 | 0.802 | 0.001 | 3.340 | 0.072 | 0.043 | 24.003 | **<0.001** | 0.150 |
| RF L | 4.147 | **0.045** | 0.053 | 11.633 | **<0.001** | 0.136 | 29.994 | **<0.001** | 0.288 |

T 1, toe 1; T 2-3-4-5, toes 2-3-4-5; MH1, metatarsal head 1; MH2, metatarsal head 2; MH3, metatarsal head 3; MH4 metatarsal head 4; MH5, metatarsal head 5; MF M, midfoot medial; MF L, midfoot lateral; RF M, rearfoot medial; RF L, rearfoot lateral.

| **Region** | **Group***  **Height** | | | **Height** | | | **Group** | | |
| --- | --- | --- | --- | --- | --- | --- | --- | --- | --- |
|  | **F (*1, 38*)** | ***p* value** | ***η_p_²*** | **F (*1, 38*)** | ***p* value** | ***η_p_²*** | **F (*1, 38*)** | ***p* value** | ***η_p_²*** |
| T1 | 0.143 | 0.706 | 0.002 | 0.618 | 0.434 | 0.008 | 0.292 | 0.519 | 0.004 |
| T 2-3-4-5 | 3.088 | 0.083 | 0.041 | 2.064 | 0.155 | 0.027 | 11.285 | **0.001** | 0.134 |
| MH1 | 2.804 | 0.098 | 0.037 | 1.121 | 0.293 | 0.015 | 11.198 | **0.001** | 0.133 |
| MH2 | 0.286 | 0.595 | 0.004 | 0.087 | 0.769 | 0.001 | 0.135 | 0.715 | 0.002 |
| MH3 | 6.464 | **0.013** | 0.081 | 0.348 | 0.557 | 0.005 | 17.940 | **<0.001** | 0.197 |
| MH4 | 1.219 | 0.273 | 0.016 | 0.001 | 0.981 | 0.000 | 49.715 | **<0.001** | 0.405 |
| MH5 | 0.001 | 0.970 | 0.000 | 0.941 | 0.335 | 0.013 | 0.257 | 0.614 | 0.004 |
| MF M | 1.191 | 0.279 | 0.016 | 0.015 | 0.904 | 0.000 | 14.443 | **<0.001** | 0.165 |
| MF L | 0.313 | 0.577 | 0.004 | 1.110 | 0.296 | 0.015 | 16.966 | **<0.001** | 0.189 |
| RF M | 1.050 | 0.309 | 0.014 | 1.158 | 0.285 | 0.016 | 7.654 | **0.007** | 0.095 |
| RF L | 1.756 | 0.189 | 0.023 | 2.848 | 0.096 | 0.038 | 4.701 | **0.033** | 0.061 |

Table S3. Results of mixed-design ANOVA of Peak Pressure

T 1, toe 1; T 2-3-4-5, toes 2-3-4-5; MH1, metatarsal head 1; MH2, metatarsal head 2; MH3, metatarsal head 3; MH4 metatarsal head 4; MH5, metatarsal head 5; MF M, midfoot medial; MF L, midfoot lateral; RF M, rearfoot medial; RF L, rearfoot lateral.
